# Compressive Fatigue Behavior of Gum and Filled SBR Vulcanizates

**DOI:** 10.3390/polym13091497

**Published:** 2021-05-06

**Authors:** Liu Yang, Lin Wang, Huaiqing Guo, Aihua Du

**Affiliations:** Key Lab of Rubber-Plastics, Ministry of Education, Qingdao University of Science & Technology, Qingdao 266042, China; 15040002331@163.com (L.Y.); 15964913857@163.com (L.W.); huaiqingguo@163.com (H.G.)

**Keywords:** styrene-butadiene rubber, fatigue, compressive stress, carbon black, creep

## Abstract

The influence of carbon black on physical mechanical properties, compressive fatigue life, and the temperature changes during compression fatigue process of styrene-butadiene rubber (SBR) vulcanizates were explored. A series of unfilled and filled SBR compounds were prepared, and the compressive fatigue behaviors of the vulcanizates were performed on a mechanical testing and simulation (MTS) machine. The top surfaces of the filled SBR were imaged using scanning electron microscopy (SEM) after 105 cycles of compressive fatigue. The filled SBR shows greater compressive fatigue resistance than the unfilled SBR. The incorporation of carbon black into SBR improves the creep resistance. The best compressive fatigue resistance for the filled SBR was achieved by the addition of 30 phr carbon black. With the increase of carbon black content, the energy dissipation and the heat build-up increase simultaneously. Furthermore, SEM images of the vulcanizates suggest that the crack propagation mechanism of the unfilled and the filled SBR was different. For the unfilled SBR, due to periodical compressive stress, the polymer chains can be destroyed, and the cracks can be easily initiated and propagated, showing serious damage on the top surfaces of the specimen. However, for the filled SBR, the carbon black agglomeration around the cracks can greatly delay the generation of the cracks, decrease the fatigue damage, and ultimately improve the fatigue resistance.

## 1. Introduction

Rubber materials can be used as vibrational dampers and shock absorbers, which are typically subjected to cyclic compressive deformations during operation. Therefore, for vibrational dampers and shock absorbers, the performance of fatigue resistance under compressive conditions is of great importance. Nowadays, few research studies focus on the compressive fatigue mechanism of styrene-butadiene rubber (SBR), which is a kind of non-crystallizable rubber and contains excellent damping behavior over a wide range of temperatures and frequencies [1,2,3]. As it is very different from natural rubber (NR), the fatigue mechanism of SBR is not dependent on the strain crystallization, so that it is very suitable for studying the fatigue mechanism [4,5,6,7]. In general, for rubber materials, a wide range of mechanical properties is realizable by proper regulation of the compound formulation, such as the type of elastomers, fillers, curatives, anti-degradants, etc. [8,9,10]. Rubber fatigue behavior is greatly affected by the properties of the filler system. It is well known that carbon black imparts excellent reinforcement to rubber, and improves the strength due to a strong interaction between rubber and filler [11,12,13,14]. The incorporation of moderate carbon black can improve energy dissipation through the movement of rubber-filler networks, and enhance the fatigue resistance of rubber [14,15,16].

Filled rubbers exhibit not only chain networks, but also filler-rubber networks. Compared with the unfilled rubber, there is not only higher energy dissipation capacity at high strains, but also more obvious viscous dissipated response under the action of medium and low strains (especially cyclic loading) for the filled rubbers. In order to extend the serve life, many studies on the fatigue behaviors of rubber materials have been reported. Abraham et al. [17] investigated the influence of minimum stress and stress amplitude on the fatigue resistance of non-crystallizable ethylene propylene diene rubber (EPDM) under load control at 1 Hz until failure. They pointed out that the mean stress is an important parameter affecting the service life for both unfilled and filled EPDM. The fatigue life increases with increasing the maximum stress, and the fatigue behavior is correlated to the properties of the rubber-filler system. Most of previous works focused on the fatigue behavior of rubber under tensile load but less attention has been dedicated to the compressive fatigue damage behaviors [18,19,20]. It is very different from the behavior under tensile conditions that the rubber materials usually do not break directly, but seriously deform or wear when they are subjected to a compressive load for a long time [21]. Chia-Chin Wu [22] measured the stress–strain response and the mechanical properties of SBR/NR vulcanizates under both static and dynamic compression conditions. Two new properties, the energy reduction factor and the softening factor, were observed and simulated during the cyclic compression test.

In the present work, we mainly concentrate on the compression fatigue behavior of SBR composites filled with different contents of carbon black. The dynamic and the static compression fatigue behaviors were investigated by hysteresis loops, dissipated energy, S-N curves, heat build-up, damage surface morphology, compression set, and creep behavior. The goal is to understand in more details of the compression fatigue mechanism of the filled SBR composites. The results in this paper will provide new insight into the difference of fatigue mechanisms in the dynamic and the static environments between unfilled and filled SBR. In the meantime, the optimum content of carbon black in SBR to resist the compressive fatigue can be obtained.

## 2. Materials and Methods

Emulsion styrene-butadiene rubber (ESBR 1502) was acquired from SINOPEC QILU company LTD; high abrasion furnace black (N330, the average size of N330 is 29 nm) was the product of Qingdao Degussa Company. Other additives, such as zinc oxide (ZnO), stearic acid (SA), the anti-aging agent N-isopropyl-N′-phenyl-p-phenylenediamine (4010NA), the accelerator N-tert-butylbenzothiazole-2-sulphenamide (NS), and sulfur (S) were industrial grade products. Detailed formulations of unfilled and filled SBR compounds are shown in Table 1. SBR 0 refers to SBR gum vulcanizate. The numbers 1–5 mentioned below represent the SBR composites with different dosage of carbon black (10, 20, 30, 40, 50 per hundred rubber, phr).

Two stages of mixing procedure were used to prepare the compounds. During the first step, SBR was masticated at 50 °C in an internal mixer (XSM-500) with a constant rotor speed of 65 rpm for 2 min, then SA, ZnO, NS, 4010 NA were put into the mixing chamber and mixed for another 3 min. After that, N330 was added into the mixer and mixed for 5 min then discharged. In the second step, the curatives were added into compound on a roller mill (SK-160B). Final compounds were stored at ambient temperature at least 10 h. Vulcanization was carried out at 160 °C with a pressure of 10 MPa.

Curing characteristics, such as lowest torque (M_L_), highest torque (M_H_), scorch time (t_10_), and optimum curing time (t_90_) of the SBR compounds were determined by a moving die rheometer (type MDR GT-M2000-A) at 160 °C.

Tensile testing was carried out with a GT-AI-7000 M universal material tester at room temperature with the tension speed of 500 mm/min according to ASTM D3039.

Compression fatigue behavior was performed on mechanical testing and simulation (MTS) machine at the amplitude of 3.75 mm, 10 Hz frequency using a sinusoidal signal. The shape and size of the testing samples are the same as the specimens of compression set (12.5*29^2^ mm). In order to reduce the residual strain resulting from the Mullin’s effect, all samples were pre-cycled at the maximum strain of 3.75 mm for 5 cycles before testing.

Heat build-up was measured by using a RH-2000N flexometer at the temperature of 55 ± 1 °C with the stroke length 5.71 ± 0.03 mm and a load of 2.00 ± 0.06 MPa. Cylindrical rubber samples of 25 mm height and 17.5 mm in diameter were used.

The surface temperatures of the samples were recorded with an IR thermography camera (FLIRONE PRO) during and after the compression fatigue experiments.

The compression set tests were performed at room temperature for 72 h according to ASTM D395-03. After relaxation at room temperature for 30 min, the height of the cylindrical samples were recorded and the compression set (C_d_) was determined using the following equation.

(1) $$C_{d}=\frac{H_{0}-H_{f}}{H_{0}-H_{s}}\times 100$$

where H_0_ is the original height of the cylindrical sample, H_s_ is the compressed height (25% of H_0_), and H_f_ is the final height of the sample.

Creep tests were also carried out on MTS when compressed the sample by a stress of 1 MPa and kept for one hour. The time-dependent deformation was finally showed.

The surface morphology of the SBR vulcanizates before and after compressive fatigue were obtained by Scanning Electron Microscope (JSM-6700F SEM).

## 3. Results

### 3.1. The Effects of Carbon Black Content on the Curing Characteristics of the Compounds

The cure characteristics of the compounds are shown in Table 2. The curing rate index (CRI) was calculated with Equation (2):

(2) $$\mathrm{CRI}=\frac{M_{H}-M_{L}}{t_{90}-t_{10}}\times 100\%$$

It was found that the filled rubber composites (1–5) showed longer optimum curing time (t_90_) than the unfilled sample. However, among these filled samples, with the increase of carbon black content, the t_90_ generally decreased. It can also be seen in all compounds that M_L_, M_H_, torque difference (M_H_-M_L_) and CRI increased with increasing the content of carbon black in the compounds. These results indicate that a positive effect on the curing rate was incorporated by the use of carbon black due to the alkaline feature of N330, thus shortened the optimum curing time.

### 3.2. The Effects of Carbon Black Content on the Mechanical Properties and Fatigue Life of the Vulcanizates

Table 3 shows the physical mechanical properties of the filled SBR vulcanizates. It can be seen that the tensile strength of all the samples increased with the increase of carbon black content. It corresponded well with the difference of the torque value (M_H_-M_L_). However, the elongation at break first increased and then decreased by the addition of carbon black. These results indicate that the SBR vulcanizate with 30 phr carbon black showed the best reinforcing properties. However, with the further addition of carbon black, the filler agglomerates increased, which resulted in a worse dispersion and a decrease of elongation at break and tear strength.

### 3.3. Cyclic Stress–Strain Behavior

**Figure 1:**
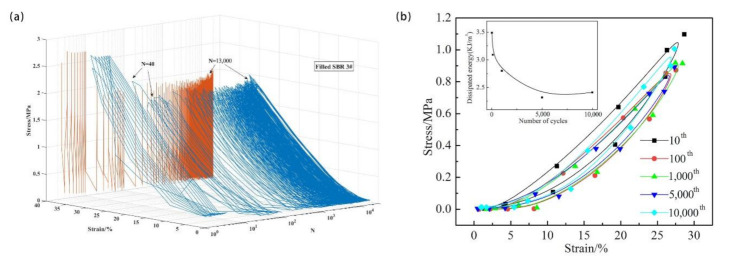
(**a**) Three-dimensional hysteresis loops of filled SBR 3, (**b**) stress–strain curves (2D hysteresis loops for several certain loading cycles), and corresponding dissipated energy of two samples during 10,000 fatigue cycling.

The compression fatigue behavior of the SBR vulcanizate is normally dependent on the cyclic loading. In this section, to reveal the damage mechanism of the unfilled and the filled SBR, three-dimensional (3D) hysteresis loops were explored and correlated with the dissipated energy. The area of the hysteresis loops equals the dissipated energy during one certain loading cycle. The experimental results indicate that the compression fatigue behavior is significantly affected by the incorporation of filler. As shown in Figure 1a, when the sample was exposed to a cyclic loading, three stages were observed in the 3D stress–strain curves. At the first stage, the maximum stress decreased greatly, which was attributed to the stress-softening effect (the Mullin’s effect). The Mullin’s effect is an initial transient softening of the stress–strain curve in rubber, especially in filled rubber. In the second stage, the stress of the sample decreased to the minimum value (N = 40) and then reached a plateau. In the third stage, the stress increased rapidly, which was regarded as compression fatigue failure of the vulcanizates, and the corresponding cycle of failure was considered the compression fatigue life of the SBR (N_f_, SBR3 = 13000). Figure 1b shows the 2D hysteresis loops of SBR 3, where the stress–strain curve can be observed more directly. It can be seen in the smaller figure that the dissipated energy decreased rapidly with the increase of the fatigue cycles. During the cyclic loading, the rubber-filler networks were partially damaged. Moreover, the degradation of the network structure caused by the crosslinking and chain scission also occurred. The damaged network structure no longer plays a role in energy dissipation, which leads to the dissipated energy decreases with the number of cycles.

**Figure 2:**
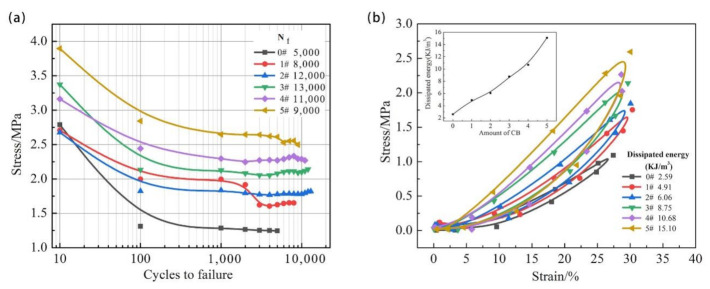
S–N curves (**a**) and stress–strain curves (**b**) of the unfilled and the filled SBR with different carbon black content.

In order to determine the effect of carbon black on the fatigue life of SBR, the data obtained by the fatigue tests were used to plot the S–N curves as shown in Figure 2a. It was observed that carbon blacks significantly prolonged the fatigue life of the composites compared with the unfilled vulcanizates. However, similar to the trends of elongation at break and tear strength, the fatigue life became shortened when the content of carbon black was more than 30 phr. Below this content, the reinforcement of the composites gradually increased by the addition of filler, which resulted in the improvement of the fatigue life, elongation at break, and tear strength. On the contrary, above the optimal content, the samples containing excessive filler were more prone to reduce the fatigue life, and some mechanical properties due to the phenomenon of carbon black agglomeration. At the same time, the dissipated energy of each cycle, which was mainly transformed into heat, was calculated by integration of the stress–strain curves, as shown in Figure 2b. It can be observed that the dissipated energy gradually increased with increasing the content of carbon black.

**Figure 3:**
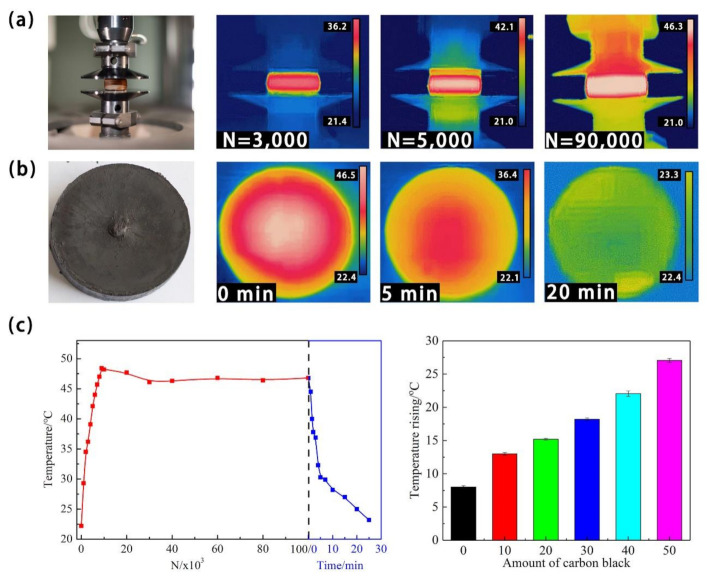
(**a**) Photo and IR images of filled SBR 3 during the compressive loading process, (**b**) photo and cooling process of SBR 3 after 10^4^ cycles, (**c**) temperature cycles/time curve of SBR 3 during the whole compression fatigue and cooling process and heat build-up properties of SBR with different carbon black content.

To examine the effect of temperature during the fatigue process on fatigue behavior of filled SBR, we took the infrared (IR) images at the number of different cycles of the filled SBR 3, during and after the compressive fatigue test (Figure 3a,b). In the IR images, we observed that the temperature in the middle part of the side surface was the highest (Figure 3a). After fatigue experiments, the top surface of SBR 0 was damaged after 10^5^ cycles of compression, especially in the central area of the top surface (the photo in Figure 3a). Moreover, the temperature distribution was observed over the top surface of the samples as shown in IR images. We can see that the temperature was the highest in the central area, which led to more serious damage in the center. Near the borders of the top surface, the temperature was lower due to the heat transfer to the surroundings. It can also be seen that the temperature of the sample rapidly dropped to room temperature within 20 min, as soon as the cyclic loadings stopped. The temperature changes during the whole compressive fatigue experiments are presented in Figure 3c. When the compression started, the temperature of the sample rapidly rose until it reached a peak value, then lightly decreased, and remained at a stable temperature. This is because of the heat exchange processes in the environment. As shown in Figure 3, due to the inner friction between carbon black and the polymer chain, the temperature rising of the SBR increased by increasing the amount of carbon black, which followed the same trend with the energy dissipation.

### 3.4. Surface Morphology before and after Dynamic Compression Fatigue

**Figure 4:**
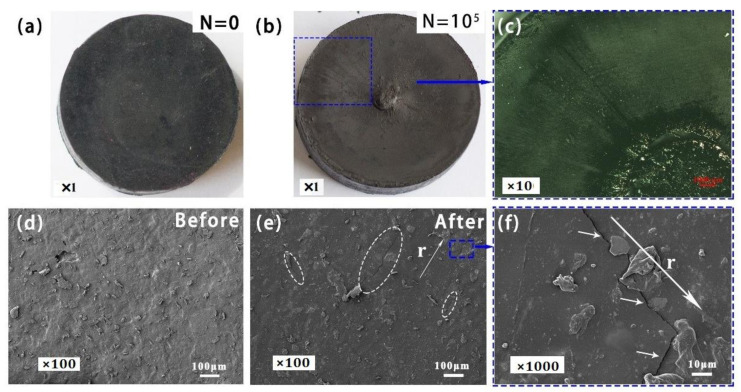
Photos and SEM images of top surface damages of filled SBR 3 before and after 10^4^ compressive test cycles. (**a**,**b**) The photo images before and after fatigue test. (**c**) Zoomed-in photo of damaged top surface. (**d**) SEM images before fatigue test. (**e**) SEM images after fatigue test. Many cracks can be observed on the top surface. (**f**) Magnified SEM images of the cracks.

The morphology of damage surface played an important role in the compression fatigue mechanism. The scanning electron microscopy (SEM) images of the damaged surface of SBR are shown in Figure 4. As shown in Figure 4a,d, the top surface of both samples before the compression fatigue tests were smooth and flat. According to our previous work [23], for unfilled SBR, there are many cavities and serious damage on the surface. However, for the filled SBR, no obvious cavities were present on the surface, whereas different damages and cracks appeared on the top surface, as shown in Figure 4b,c. After 10^5^ compressive cycles, it can be clearly seen that the damage was shown on the surface, and massive cracks appeared on the top surface, as shown in Figure 4e,f. As stated above, the mechanical properties of the unfilled sample were inferior to that of the filled samples. Thus, with increasing the compression fatigue cycles, the stress would directly induce the break of the chain networks, resulting in serious crack propagation. However, the damage of the vulcanizates apparently alleviated after the incorporation of the fillers. For the filled SBR, there were many filler particles in the rubber, which hindered crack propagation and, finally, improved the compressive fatigue resistance of the filled SBR. As shown in Figure 4f, it can be clearly seen that the propagation path of the crack was changed by filler particles.

**Figure 5:**
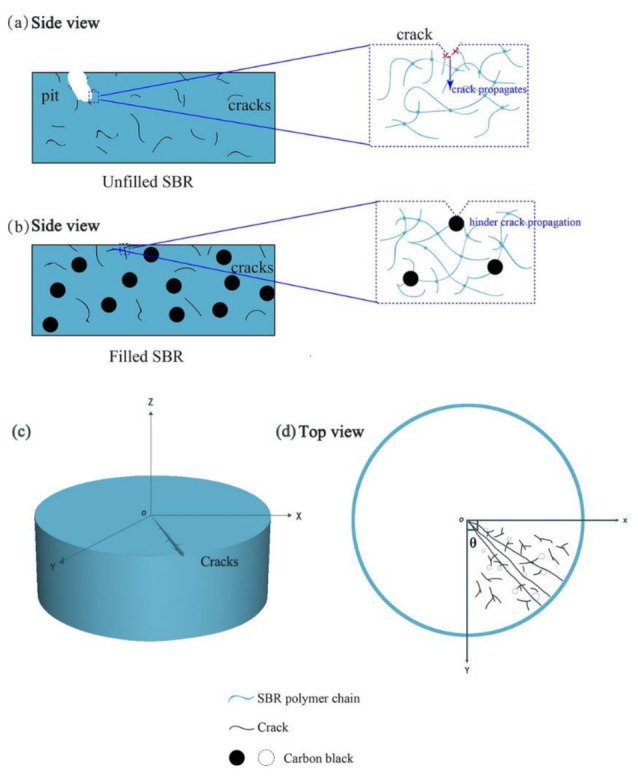
Schematic of fatigue damage in rubber caused by cyclic compressive loading: (**a**) unfilled SBR, (**b**) filled SBR, (**c**) cracks, and (**d**) top view on the top surface.

Figure 5 illustrates the different characteristics of fatigue behaviors of the unfilled and the filled SBR caused by periodical compressive stress. It is obvious that compression stress induced cracks from the subsurface to the interior. With the further increase of the compressive stress cycles, cracks extended and propagated, and finally led to surface damage. However, the crack propagation mechanisms of the unfilled and the filled SBR were different. For the unfilled SBR, due to the periodical compressive stress, the polymer chains were destroyed, and the cracks were easily initiated and propagated, resulting in many holes on the surface, as shown in Figure 5a. For the filled SBR, the carbon black around the cracks greatly reinforced the crack tips, suppressing the generation of the cracks, changing the propagation path of the crack, and prolonging the fatigue life (Figure 5b). Finally, the top surface showed obvious cracks, as shown in Figure 5c,d.

### 3.5. Compressive Creep Behavior

**Figure 6:**
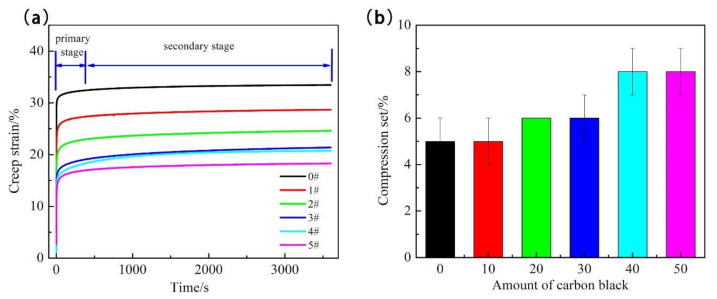
Creep behavior (**a**) and compression set (**b**) of unfilled and filled SBR.

The static and the dynamic mechanical properties of rubber material usually interact with each other. The time dependency of the mechanical properties is related to the creep behavior when it is subjected to a sustained stress. Therefore, in this section, the compressive creep behavior and the compression set are discussed in order to investigate the monotonic compressive behavior. The creep behavior of the vulcanizates is shown in Figure 6a. The curves were divided into two stages, the primary, and the secondary creeps. The creep strain sharply increased in the primary stage, and then remained constant in the secondary creep. It can be seen that creep strain in the primary stage dominated nearly the total creep strain during 3600s. In addition, the unfilled SBR had higher creep strain than the filled SBR vulcanizates, and the magnitude of the creep strain decreased with the increase of carbon black at the same testing time. Therefore, carbon black provides excellent creep resistance. This is substantially consistent with the results of the dynamic compressive experiments. With the moderate increase of the carbon black content, the creep resistance improved and the fatigue life extended, simultaneously. The mobility of molecular chains is limited by the filler-rubber interaction, which improves the creep resistance.

Unlike the results of the creep tests, the values of permanent deformation increased with the increase of filler content, as shown in Figure 6b. The compression set of unfilled SBR was 5%, which was much lower than the compression set 8% of filled SBR with 50 phr of carbon black. That means, after the external force was removed, the deformation of the sample was more difficult to recover for the high carbon black loaded compound, resulting in permanent deformation. This phenomenon induced by the addition of carbon black limits the mobility of polymer molecular chain, which makes it difficult to recover from deformations. Although the creep strain decreases and the permanent deformation increases with the increase of the amount of carbon black, there is an optimal amount of carbon black.

## 4. Discussion

SBR gum vulcanizates and filled SBR vulcanizates were prepared by the addition of different contents of carbon black. The mechanical properties, dynamic compressive fatigue behaviors, and mechanisms of the SBR composites were studied. The main conclusions are summarized as follows:

(1) Along with increasing the carbon black content, the curing rate index (CRI) increases, which means carbon black has a positive effect on the curing rate. When the amount of carbon black is 30 phr, SBR vulcanizates show the best reinforcing properties. The magnitude of creep strain decreases with an increase of carbon black content, but the compression set of all the samples increases with increasing filler content.
(2) When the samples are exposed to cyclic loading, the maximum stress is reduced due to the stress-softening effect at the first stage. At the second stage, the maximum stress decreases sharply and then reaches a plateau. Finally, maximum stress increases again, which is regarded as N_f_. It is observed that carbon black can prolong the N_f_. However, the content of carbon black has an optimal value of 30 phr.
(3) The dissipated energy and the temperature rising gradually increases with increasing the content of carbon black. Since the temperature is difficult to transfer, the temperature in the middle part of the sample is the highest.
(4) For filled SBR, massive cracks appear on the top surface. However, with the content of carbon black increasing, the damage of the vulcanizate alleviates.

## Figures and Tables

**Table 1:** Formulations in parts per hundred rubber (phr).

Ingredients	ESBR	ZnO	SA	4010 NA	NS	S	N330
Unfilled SBR	100	5	2	2	1	2	-
Filled SBR	100	5	2	2	1	2	Variable (10,20,30,40,50)

**Table 2:** Cure characteristics of unfilled and filled SBR.

Samples	M_L_/dNm	M_H_/dNm	t_10_/min	t_90_/min	M_H_-M_L_/dNm	CRI/dNm·min^−1^
0	0.59	8.8	6.52	13.94	8.21	1.11
1	0.37	23.37	7.40	27.53	23.00	1.14
2	1.32	26.99	7.18	23.38	25.66	1.58
3	2.31	30.84	6.73	23.22	28.53	1.73
4	3.00	33.96	5.47	20.73	30.96	2.03
5	3.87	37.32	4.52	20.78	33.45	2.06

**Table 3:** Mechanical properties of unfilled and filled SBR vulcanizates.

Samples	0	1	2	3	4	5
Tensile strength/MPa	1.72 ± 0.19	6.24 ± 1.29	11.62 ± 0.73	20.22 ± 0.67	21.93 ± 1.08	23.16 ± 0.57
Elongation at break/%	367 ± 32	436 ± 52	458 ± 4	502 ± 8	436 ± 10	420 ± 9
Tear strength/kN·m^−1^	7.64 ± 1.30	11.59 ± 2.17	22.99 ± 4.87	23.59 ± 3.63	32.08 ± 4.41	27.99 ± 2.37

## Data Availability

Not applicable.
